# Effect of Common Buffers and Heterocyclic Ligands on the Binding of Cu(II) at the Multimetal Binding Site in Human Serum Albumin

**DOI:** 10.1155/2010/725153

**Published:** 2010-05-05

**Authors:** Magdalena Sokołowska, Krystyna Pawlas, Wojciech Bal

**Affiliations:** ^1^Department of Hygiene, Wrocław Medical University, Mikulicza-Radeckiego 7, 50-345 Wrocław, Poland; ^2^Institute of Biochemistry and Biophysics, Polish Academy of Sciences, Pawińskiego 5a, 02-106 Warsaw, Poland; ^3^Central Institute for Labour Protection-National Research Institute, Czerniakowska 16, 00-701 Warsaw, Poland

## Abstract

Visible-range circular dichroism titrations were used to study Cu(II) binding properties of Multimetal Binding Site (MBS) of Human Serum Albumin (HSA). The formation of ternary MBS-Cu(II)-Buffer complexes at pH 7.4 was positively verified for sodium phosphate, Tris, and Hepes, the three most common biochemical buffers. The phosphate > Hepes > Tris order of affinities, together with strong spectral changes induced specifically by Tris, indicates the presence of both Buffer-Cu(II) and Buffer-HSA interactions. All complexes are strong enough to yield a nearly 100% ternary complex formation in 0.5 mM HSA dissolved in 100 mM solutions of respective buffers. The effects of warfarin and ibuprofen, specific ligands of hydrophobic pockets I and II in HSA on the Cu(II) binding to MBS were also investigated. The effects of ibuprofen were negligible, but warfarin diminished the MBS affinity for Cu(II) by a factor of 20, as a result of indirect conformational effects. These results indicate that metal binding properties of MBS can be modulated directly and indirectly by small molecules.

## 1. Introduction

Human serum albumin (HSA) is the most abundant protein of blood serum, at a concentration of ca. 0.6 mM (~4%). It is a versatile carrier protein, involved in the transport of hormones, vitamins, fatty acids, xenobiotics, drugs and metabolites [1]. HSA also carries metal ions, including physiological Ca^2+^, Zn^2+^, Co^2+^, and Cu^2+^, as well as toxic Cd^2+^ and Ni^2+^ [1–3]. Four distinct metal binding sites have been characterized so far in HSA. Cys-34, the only free thiol in HSA, is located at the bottom of a hydrophobic pocket. It selectively binds hydrophobic complexes of heavy metal ions, such as Pt^2+^ and Au^+^ [4–7]. Three other sites are more accessible and more versatile. The N-terminal binding site (NTS, also called the ATCUN motif) has been characterized particularly well. It is composed of the first three amino acid residues of the HSA sequence, Asp-Ala-His. This sequence is not confined sterically, as evidenced by the absence of trace of these residues in X-ray structures of HSA [1, 2] and has a conformational freedom to wrap around a metal ion. The resulting square-planar complex exhibits a rare coordination mode with deprotonated amide nitrogens of Ala and His residues, in addition to the N-terminal amine and the His-3 imidazole donor [8–10]. It provides the primary binding site for Cu(II) and Ni(II) ions and the tertiary site for Co(II) ions [10, 11]. We recently demonstrated that the conditional dissociation constant for Cu(II) binding at the NTS is as low as 1 pM [12]. The studies of short peptide models of similar His-3 sites performed in our and other laboratories, for example, by Hadjiliadis et al., uniformly indicated their very high affinities for Cu(II), and also Ni(II) ions [13–15]. There is abundant experimental evidence for the complete saturation of the coordination sphere of the Cu(II) ion bonded to NTS and model peptides [9, 11–16].

Another site is composed of side chains of His67, His247, Asn99 i Asp249. It is located at the interface of domains I and II of HSA. We named it multimetal binding site (MBS) in our previous study, because we demonstrated that it can bind Cu(II), Ni(II), Zn(II), Cd(II), as well as Co(II) ions with comparable affinities [10, 11]. This site is identical with cadmium site B (CdB) characterized by NMR spectroscopy [17–19]. Recent studies on Zn(II) binding at MBS provided crucial data about its spatial arrangement [18]. The location of the third site, called cadmium site A (CdA), remains unknown. It is the primary site for Cd(II) and Co(II) ions but does not participate in Cu(II) binding [11]. 

The structure of MBS was analyzed by NMR and molecular modeling of the Zn(II) complex [18]. The Zn(II) ion is pentacoordinate in a square pyramidal geometry, with equatorial coordination provided by two imidazole nitrogens (His67 and His247), a carboxylate oxygen (Asp249) and a carbonyl oxygen (Asn99). The axial site in this structure is occupied by a water molecule. This fact suggests that the MBS binding site may be modified by external ligands, forming hypothetical ternary species. 

In this work we attempted to find out whether common buffers may interact with the Cu(II) ion bonded at MBS. We also tested whether occupation of hydrophobic pockets, which are used for transportation of endogenous and exogenous aromatic molecules [20–23], may affect the coordination properties of MBS.

## 2. Experimental

### 2.1. Materials

Homogeneous, high purity defatted HSA was obtained from Sigma. CuCl_2_, Hepes, Tris, sodium phosphate, and NaCl were obtained from Aldrich. Stock solution of CuCl_2_ was standardized complexometrically. CuCl_2_ solutions were calibrated as described in [15]. NaOH and HCl were purchased from Merck. 

### 2.2. Methods

The CD spectra were recorded at 25°C on a Jasco J-715 spectropolarimeter (JASCO, Japan Spectroscopic Co., Hiroshima, Japan), over the range of 300–800 nm, using a 1 cm cuvette. The spectra are expressed in terms of Δ*ε* = *ε*_*l*_ − *ε*_*r*_, where *ε*_*l*_ and *ε*_*r*_ are molar absorption coefficients for left and right circularly polarized light, respectively. In buffer experiments the solutions contained 100 mM Hepes, Tris, Sodium Phosphate, or NaCl, at pH 7.4. 

HSA was dissolved at 0.3–0.5 mM in either a 100 mM Hepes, Tris, Sodium Phosphate buffers, pH 7.4, or in an unbuffered 100 mM NaCl solution. The pH of both types of samples was controlled to remain in the 7.38–7.42 corridor and adjusted with submicroliter amounts of concentrated HCl or NaOH when necessary. Concentrations of stock solutions of HSA were estimated spectrophotometrically at 279 nm [9] and by Cu(II) titrations. The fractional abundance of NTS in HSA was measured by careful CD titrations using calibrated Cu(II) solutions. The average value of 0.73 was obtained, in agreement with the previous studies [10–12, 15, 24].

## 3. Results and Discussion

### 3.1. The Effect of Buffers on the Binding of Cu(II) Ions at MBS

We chose sodium phosphate, Tris and Hepes as the most common substances used for buffering protein solutions at pH 7.4. Their effect on Cu(II) binding to HSA was studied at a 0.1 M concentration stage versus unbuffered 0.1 M NaCl, used as a control solution. The choice of the latter was dictated by the presence of 0.1 M chloride in the blood serum [25]. The pH of the NaCl samples was controlled by additions of small quantities of concentrated HCl or NaOH solutions, when necessary. This was achieved thanks to the self-buffering properties of HSA, based on the presence of multiple His residues on its surface [9, 26]. The titrations were monitored by CD spectroscopy, analogously to our previous studies [10, 15]. 

**Figure 1:**
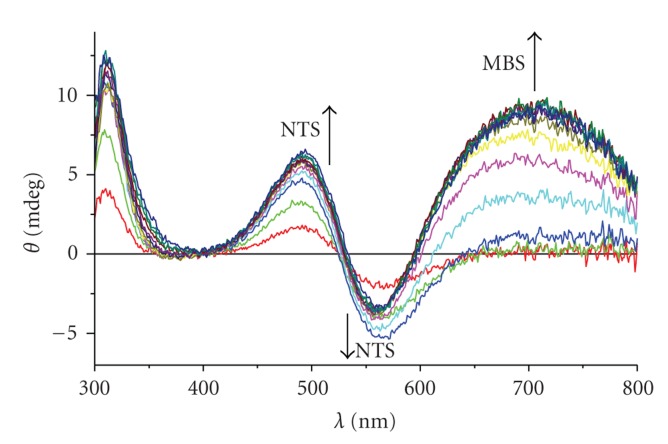
CD spectra of Cu(II) titration of HSA (0.5 mM in 0.1 M NaCl, pH 7.4), from 0 to 3 mol equivalents, at a 0.25 mol equivalent step.

**Figure 2:**
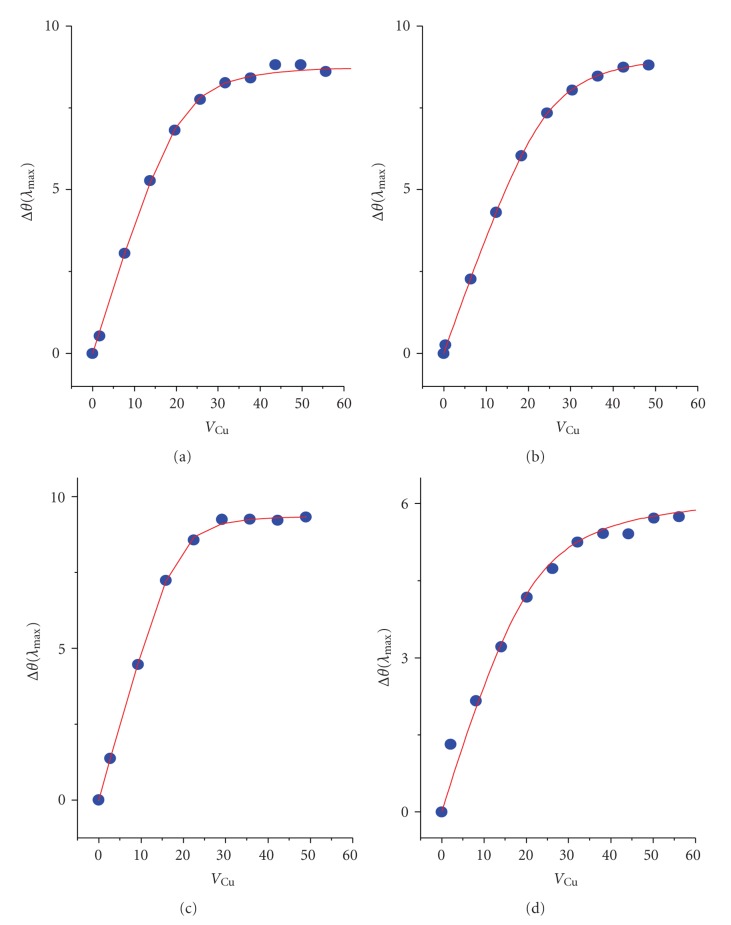
Titration curves (experimental points, 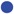, and fits to the *K*_app_ formula, 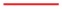) for Cu(II) binding at MBS in four solutions studied: (a) NaCl; (b) Hepes; (c) sodium phosphate; (d) Tris.

A typical titration is presented on Figure 1. Figure 2 shows titration curves, obtained from titration experiments by plotting the changes of ellipticity (Δ*θ*) at *λ*_max_ of Cu(II) complexes at MBS (665 nm for Tris and 695 nm for remaining solutions). The changes of ellipticities of transitions related to Cu(II) complexes at NTS, at 492 and 565 nm, were linear up to the NTS saturation, in accordance with a very high affinity of this site [12]. 

**Figure 3:**
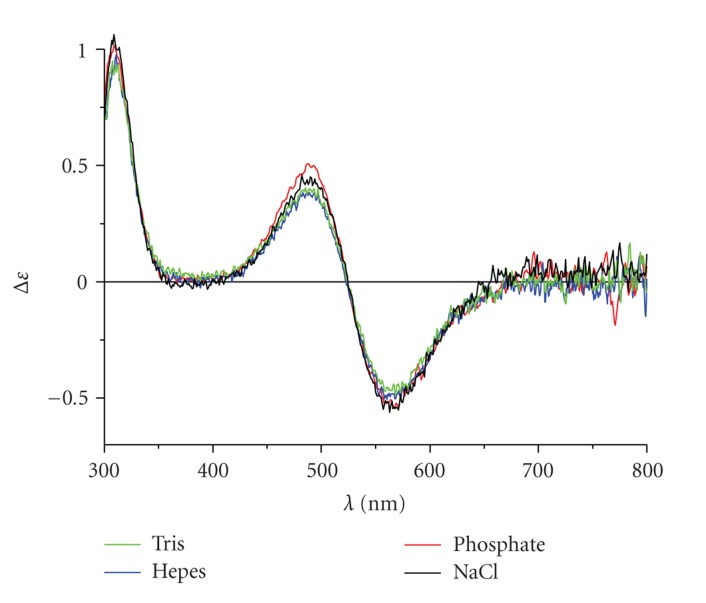
Spectra of pure forms of NTS-Cu(II) complexes obtained by extrapolation of titration data.

**Figure 4:**
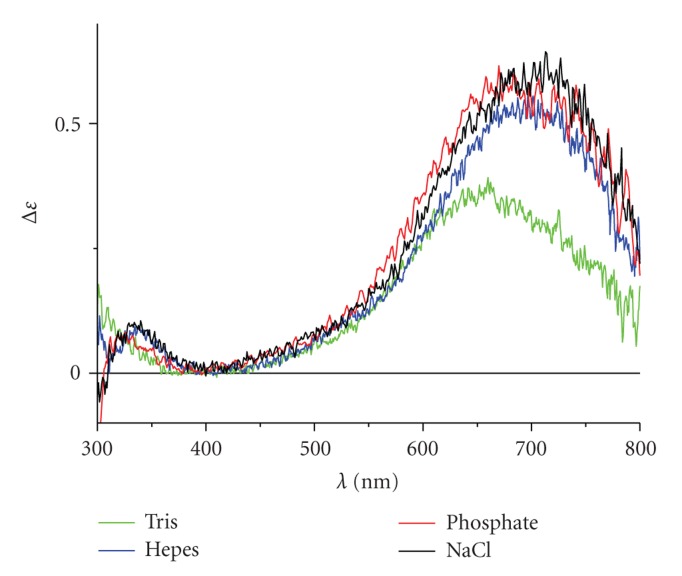
Spectra of pure forms of MBS-Cu(II) complexes obtained by extrapolation of titration data.

The pure spectra of NTS and MBS complexes in individual solutions, calculated by extrapolation of titration data, are shown in Figures 3 and 4, respectively. The shapes and intensities of NTS bands were not affected by buffers studied. In contrast, the presence of Tris altered the appearance of the characteristic MBS band considerably, compared to the NaCl reference. The effects of phosphate and Hepes were less pronounced, but still significant. 

Apparent binding constants (*K*_app_) for Cu(II)-MBS complexes in the presence of buffers were obtained from titrations presented in Figure 2. These constants, calculated on a technical assumption of the formation of binary species only, are presented in Table 1. Table 1 contains also further constants, calculated relative to the Cu(II)-MBS interaction in NaCl, taken as corresponding to a 100% binary complex formation. These calculations were prompted by spectral effects described above and by significant differences among log *K*_app_ values. 

**Figure 5:**
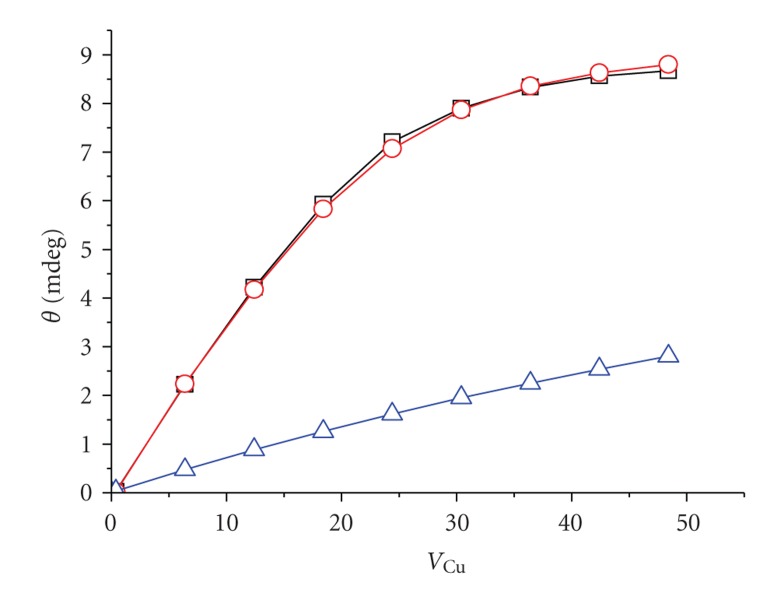
Comparison of experimental data (□), the best fit to the ternary complex model (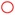), and the simulation of titration in the absence of ternary complex formation (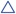) for the titration of MBS in 0.1 M Hepes, pH 7.4.

First, the effects of competition of buffers for Cu(II) were accounted for Literature protonation and Cu(II) binding constants of Hepes [27], Tris [28], and phosphate ions [29] were used in these calculations. The competition-only model of interaction, could not, however, reproduce the titration curves, and it was necessary to include ternary complex formation. Figure 5 illustrates this issue for the case of Hepes buffer. The shift of the MBS *d*→*d* band to higher energies by 30 nm, from 695 on 665 nm, also provided an unequivocal piece of evidence in favor of ternary complex formation with Tris. Calculations based on this assumption, performed for all three buffers, yielded equilibrium constants, given in Table 1. The reactions defining these constants are provided in the footnotes of Table 1. 

Briefly, *K*_111_ describes the overall formation of a ternary species, *K*_11_ is the literature binding constant of the Cu(II) complex of a buffer, *K*_e_ corresponds to the ternary complex formation by addition of buffer-complexed Cu(II) to MBS, and *K*_T_ is the equilibrium constant of the formation of the ternary complex in the reaction of two binary complexes, thus featuring the tendency of binary components to form a ternary species [33]. All these constants, except for *K*_11_, are conditional in respect to the pH value of 7.4. 

Calculations of titrations in these buffers demonstrated that the observed spectra were practically pure spectra of mixed complexes. The participation of the binary MBS complex was less than 1.5% at all titration points. This feature allowed us to speculate about the binding modes in ternary complexes. The spectra for Hepes and phosphate were practically identical with those of the binary complex, but the titration curves demonstrated the significant quantitative deviation from the binary complex-only binding. Therefore, the most likely binding mode for these two substances is that based on a substitution of the axial water molecule with a buffer oxygen donor. The comparatively high binding constants for ternary complexes, in relation to binary complexes, demonstrated the creation of accessorial interactions between HSA and the ligand. The blueshift and change of the band intensity for Tris suggests a substitution of an equatorial oxygen, perhaps that of Asn99. 

CI values presented in the last column of Table 1 were calculated for total concentrations for Cu(II) and the formal competitor *Z* = 1 *μ*M and for HSA = 0.5 mM at pH = 7.4. These calculations were done in two versions—for L (buffer) concentrations of 0.5 mM and 100 mM. These concentrations of HSA and Cu(II) ions corresponded to physiological values, the latter to the exchangeable pool of Cu(II) in blood plasma [32]. From these calculations, one can infer that the formation of ternary complexes with buffers had a relatively small impact on the affinity of Cu(II) ions to MBS. The values of *K*_*T*_ demonstrated that Hepes and Tris buffers somewhat destabilized this interaction, and phosphate ion showed a stabilizing effect. However, their effects on Cu(II) binding were significant at practically used buffer concentrations of 100 mM. Hepes was applied in successive investigations, because this buffer showed the smallest interference with Cu(II) binding parameters of MBS. 

### 3.2. Interaction between MBS and Hydrophobic Pockets for Aromatic Molecules

**Figure 6:**
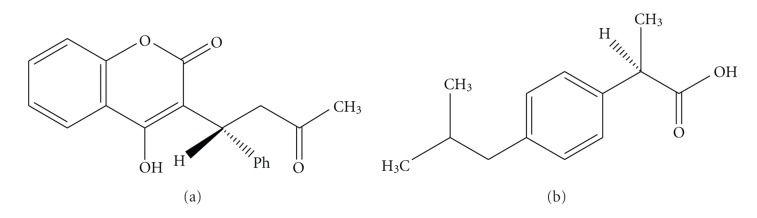
Chemical structures of warfarin (War, left) and ibuprofen (Ibu, right).

It was interesting to find out whether the binding of warfarin (War) and ibuprofen (Ibu) Figure 6, exogenous ligands characteristic for hydrophobic pockets (Sudlow's sites) I and II in HSA, respectively, could influence Cu(II) binding at NTS, and, in particular MBS. 

In these experiments we used HSA samples containing 1.75 mol equivalents of Cu(II) ions. According to the results and discussion presented above, these samples contained fully saturated NTS and one mole equivalent of Cu(II) in an equilibrium with MBS, at about 80% saturation. The samples were titrated separately with War and Ibu. None of these two substances possesses donor groups, which would can effectively bind Cu(II) ions in solution. This notion was confirmed by the absence of complex formation fingerprints in UV-vis spectroscopy and circular dichroism (CD) spectra (data not shown), and by the literature [20–22, 34, 35]. 

**Figure 7:**
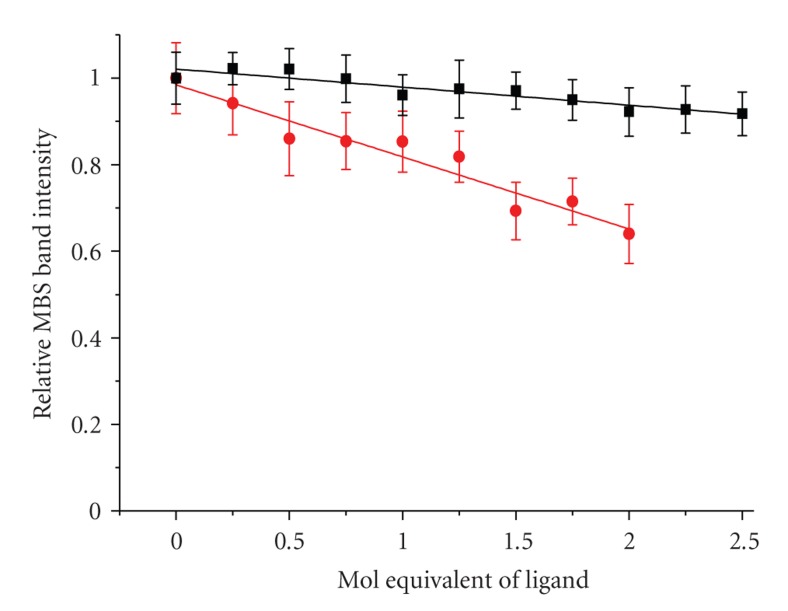
Titrations of HSA in the presence of 1.75 mol equivalents of Cu(II) with War (A) and Ibu (B) from 0 to 2.5 mol equivalents in 0.25 mol equivalent steps. The titration of War was terminated at 2 mol equivalents due to the loss of solution transparency. The solid lines represent linear fits to the data.

The HSA binding constants for War (3.3 × 10^5^ M^−1^) and Ibu (2.7 × 10^6^ M^−1^) [30, 36–38] are sufficiently high to assure stoichiometric binding to their respective binding pockets in 0.5 mM HSA solutions—the addition of one equivalent of ligand yields a 93% saturation for War and a 99% saturation for Ibu. Figure 7 shows the titrations of 0.5 mM HSA with War and Ibu in the presence of 1.75 mol equivalents of Cu(II) ions. In the course of these titrations, we observed a specific decrease of intensity of the MBS band. The band position remained unchanged. The effect of Ibu was very subtle, but that of War was clearly pronounced. Interestingly, the linearity of the band intensity decrease was similar before and after the stoichiometric binding point for both HSA ligands. 

**Figure 8:**
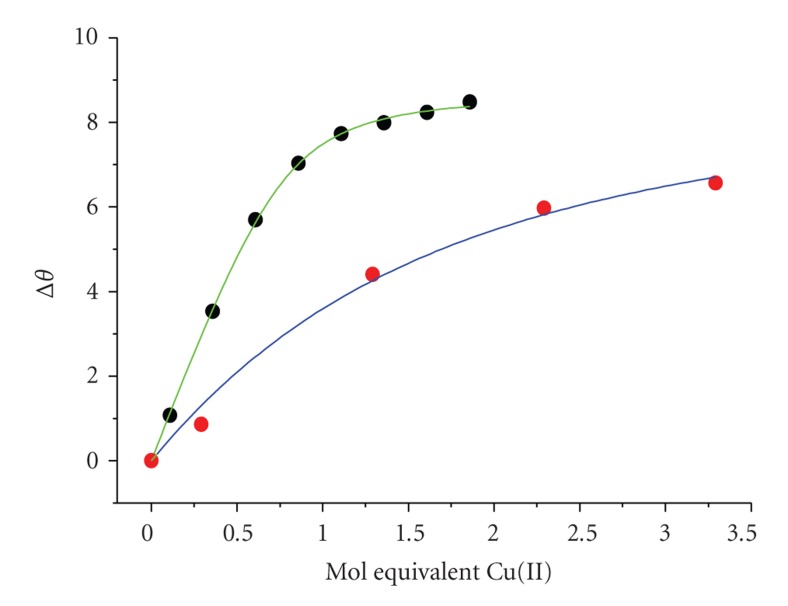
Comparison of titration curves for Cu(II) binding at MBS obtained in the presence of 1 mol equivalents of Ibu (experimental points, 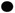, and fits to the *K*_app_ formula, 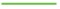) and War (experimental points, 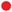, and fits to the *K*_app_ formula, 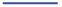).

Figure 8 presents titrations of HSA with Cu(II) ions in the presence of stoichiometric (1 mol equivalent) amounts of War and Ibu, performed in a 100 mM Hepes buffer, pH 7.4. The Ibu titration yielded the log *K*_app_ value of 4.49(5), identical within the experimental error with that obtained in 100 mM Hepes in the absence of Ibu (4.45(5), Table 1). The presence of War had however a pronounced effect on Cu(II) binding. A very low log *K*_app_ value of 3.2(2) was obtained. Therefore, the binding of War decreased the affinity of MBS for Cu(II) by a factor of 20. The Cu(II) binding at NTS was unaffected in both cases. 

The effects presented in Figures 7 and 8 can be interpreted by taking into account the locations of hydrophobic pockets. War binds specifically at site I, which is located in subdomain IIA of HSA, while Ibu binds specifically at site II, which is located in subdomain IIIA [1, 2, 31]. MBS is located at the interface of domains IB and IIA, much closer to site I than to site II, which is separated from MBS by a very large crevice [32]. Based on the present data, we can speculate that the binding of warfarin induced a conformational change which decreased the accessibility of MBS. The absence of CD spectral changes of the Cu(II)-MBS complex indicated, however, that the geometry of the site itself was not affected.

## 4. Conclusion

The results presented above clearly indicate that the Cu(II) binding properties of MBS of HSA can be influenced by external ligands. The order of buffer affinities for the formation of ternary MBS-Cu(II)-Buffer complexes, phosphate > *H* *e* *p* *e* *s* > *T* *r* *i* *s*, indicates that the interaction is not limited to the binding with the metal ion. This notion is supported by strong spectral changes induced specifically by Tris, which clearly indicate the addition of the Tris nitrogen atom to the coordination sphere of MBS-bound Cu(II) ion. The indirect effect of warfarin indicates a specific negative control of MBS binding of metal ions by site I ligands.

MBS is unlikely to carry Cu(II) physiologically but is considered as a major transport site for Zn(II) ions in blood plasma [18]. The interactions described in this paper are very likely to be shared by MBS occupied by a Zn(II) ion, because Cu(II) and Zn(II) binding modes are thought to be very similar. Therefore, Zn(II) binding properties of MBS may be modulated directly and indirectly by small molecules. This physiologically important issue will be investigated in our future studies.

## Figures and Tables

**Table 1:** Quantitative description of ternary complexes formed by Cu(II) ions at MBS of HSA at pH 7.4. Standard deviations of significant values of experimental logarithms constants on the last digits are given in parentheses.

Ligand (L)	log *K*_app_	log *K*_111_^a^	log *K*_11_^b^	log *K*_e_^c^	log *K*_T_^d^	CI^e^	
[L] =						0.5 mM	100 mM
NaCl	4.60(3)					7.62	
Hepes	4.45(5)	7.54(5)	3.22	4.32	−0.28	7.71	9.27
Tris	4.23(6)	8.22(5)	4.05	4.17	−0.43	7.74	9.42
Phosphate	5.17(7)	8.2(1)	3.20	5.00	+0.40	8.04	10.13

^a^ *K*_111_ is the equilibrium constant of the reaction Cu^2+^ + L + MBS = Cu(MBS)(L).

^b^ *K*_11_ is the equilibrium constant of the reaction Cu^2+^ + L = Cu(L); literature values [30–32].

^c^ *K*_e_ is the equilibrium constant of the reaction MBS + Cu(L) = Cu(MBS)(L); log *K*_111_ = log *K*_11_ + log *K*_e_.

^d^ *K*_T_ is the equilibrium constant of the reaction Cu(MBS) + Cu(L) = Cu(MBS)(L) + Cu^2+^.

log *K*_T_ = log *K*_111_ − log *K*_11_ − log *K*_app_ (NaCl).

^e^CI is the competitivity index (description in text), calculated for following total concentrations: Cu(II) and Z = 1 *μ*M, HSA = 0.5 mM, buffer = 0.5 mM, or 100 mM.
